# Celluloid

**Published:** 1878-03

**Authors:** 


*Celluloid.*—DR. C. STODDARD SMITH, the agent of the Celluloid Company, was in Baltimore a few days ago, and gave a lecture before the class of the Baltimore College of Dental Surgery, on Celluloid, bringing out some new points of great interest to those working that material, and enforcing those already known. He claims that Celluloid is greatly and wrongly abused by those not familiar with peculiarities, and that it is often a failure by reason of its working not being understood, and its remarkable poperties taken advantage of.

All apparatus which involves the use of dry heat he condemns as injurious to the plate, it being essential that the material be heated in a bath of some sort,—steam, oil or glycerine being equally good. Dry heat is injurious by reason of the fact that it heats the plate unequally.

Another point of importance is that the plate should be heated at a temperature of at least 270°. Plates may be moulded into shape at a lower temperature, but it requires the above mentioned heat to overcome the tendency of the celluloid to crawl, and to set it in the form it is desired to keep.

A third point of great importance is that the plate be kept under *continuous pressure* from the time it begins to soften. Unless this is done,—if it is allowed to expand or swell,—it can never be restored to its original solidity, and although a plate sound in appearance may be made; it cannot be depended on.

It is not demonstrated, Dr. S. thinks, that Celluloid welds perfectly, although it has the appearance of so doing in many cases. It is best not to depend on this ascribed property. The Celluloid Company invite correspondence with dentists, and solicits their co-operation in developing this substance. Any hints or suggestions will be thankfully received by them. They wish particularly to know if the older plates, which were of pale color (due to the incorporation of a little oxide of zinc) are thought to be better in consequence of the admixture of the small amount of mineral matter thus incorporated. H.

